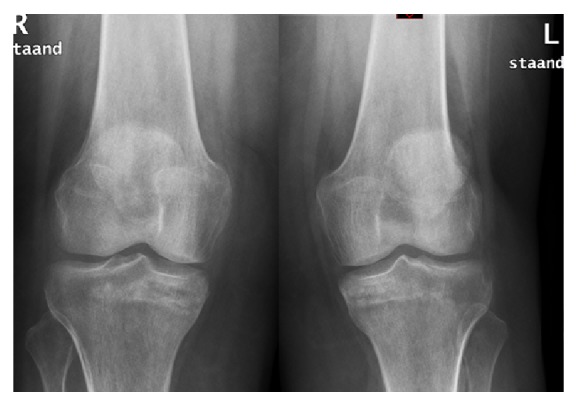# A Woman with Rheumatoid Arthritis and a Bilateral Fracture of the Proximal Tibia

**DOI:** 10.1155/2016/5094906

**Published:** 2016-02-11

**Authors:** J. Th. (Arjan) Hooghof, Joris J. Mellema, Marcel D. Posthumus, Jos J. A. M. van Raaij

**Affiliations:** Department of Orthopaedic Surgery, Martini Hospital, Van Swietenplein 1, 9728 NT Groningen, Netherlands

## Abstract

A 52-year-old woman presented herself with pain on the medial sides of the proximal tibia after a minimal trauma. Conventional X-rays did not show any pathology. However, the MRI showed a bilateral fracture of the proximal tibia. Since the patient was treated with methotrexate due to rheumatoid arthritis, methotrexate osteopathy was considered. Long term treatment with low doses of methotrexate proved to inhibit osteoblast proliferation and may eventually lead to decreased bone formation and osteopenia. On the other hand, immobilization, joint deformities, and steroid treatment are associated with rheumatoid arthritis and are also known risk factors for fractures. The clinical relevance of methotrexate osteopathy still has to be established. However, if a patient treated with methotrexate localizes pain in the tibia, methotrexate osteopathy should be considered. Withdrawal of the drug may improve symptoms.

## 1. Introduction

The incidence of knee injury in general practice in The Netherlands is 13.7 per 1000 patients per year [1]. Knee injuries occur mainly in sports, at work, or in traffic. In the acute phase fractures and neurovascular injury should be excluded. Stress fractures are common and are described in athletes and soldiers with an incidence of 5–30% [2]. In case of a stress fracture there is a mismatch between the strength of the bone and the amount of mechanical stress. It is mainly found in weight-bearing bones such as the tibia, metatarsals, and calcaneus.

Stress fractures are classified into two groups: fatigue and insufficiency fractures. A fatigue fracture is a type of stress fracture that is the result of abnormal or chronic repeated stress on normal bone. The “march fracture” of the metatarsals is well known. An insufficiency fracture is a type of stress fracture which is the result of normal load on abnormal bone and is especially seen in osteoporosis. Common sites are the vertebrae, tibia (both proximal and distal), the sacrum, and the femur. The clinical presentation of knee injuries may be misleading as is described in the case history of a patient.

## 2. Case History

A 52-year-old woman presents herself at the orthopedic outpatient department with progressive pain in both knees since one year. The pain had an acute onset after falling over a low garden fence. The pain is continuously present and is located on the medial sides of the knees. No joint effusion, instability, or locking sensations were noticed. The patient was mostly affected by a significantly reduced walking distance. There was no night pain. Knee stiffness was felt throughout the day. Physical therapy reduced this stiffness, but the pain persisted. Medical history mentions rheumatoid arthritis for 10 years, for which she was treated with methotrexate (MTX) 25 mg once a week and prednisone 5 mg daily.

On physical examination, there were no signs of active rheumatoid arthritis of the knees. There was no visible joint effusion. The patient had a normal posture with slight genu varum. Normal range of motion of both knees was measured. The knee ligaments were all stable. McMurray's test was positive in both knees. Pain was localized on the medial side of both knees at the proximal tibia.

Because of the medical history and typical presentation, medial meniscus injury was suspected. The pain had an acute onset after a trauma and is localized on the medial sides of the knees and McMurray's tests were positive. However, contusion, chondromalacia, osteoarthritis, and active rheumatoid arthritis could not be excluded. Further diagnostic procedures were required to be able to differentiate between these diagnoses.

**Figure 1:**
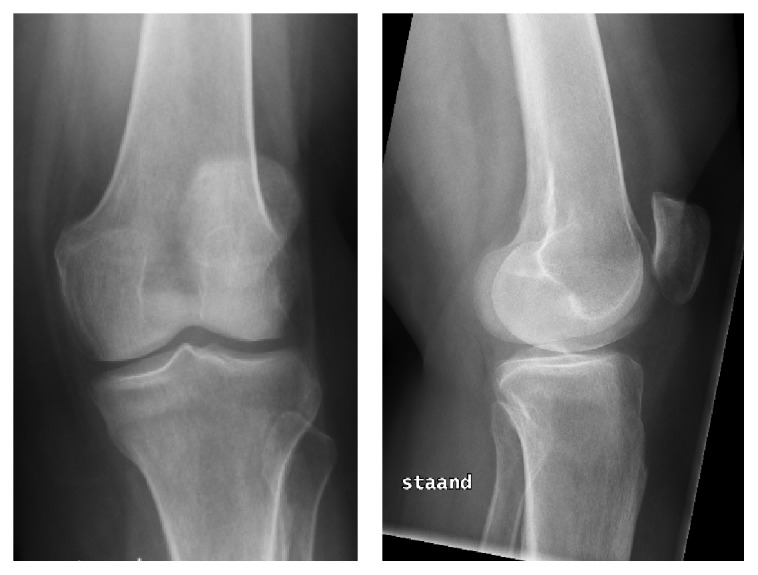


**Figure 2:**
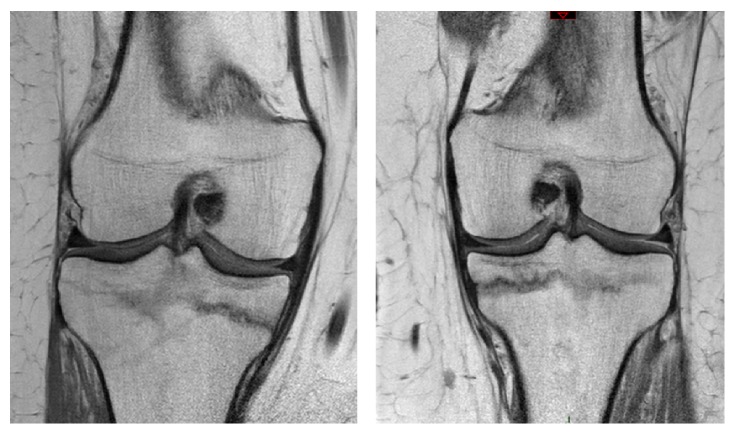


X-rays of both knees showed a normal aspect of the bone, with no evidence of a fracture, osteoarthritis, or malignancy (Figure 1). To exclude soft tissue injury magnetic resonance imaging of both knees was requested. MRI of the right knee showed intact ligaments and menisci. An interruption of the medial cortex of the proximal tibia was seen with no displacement. The horizontal fracture line does not extend to the intra-articular surface of the joint and respects the dorsal and lateral cortex of the tibia. Edema is visible around the fracture. There are no signs of collapse of the tibial plateau. MRI of the left knee shows an identical image (Figure 2).

**Figure 3:**
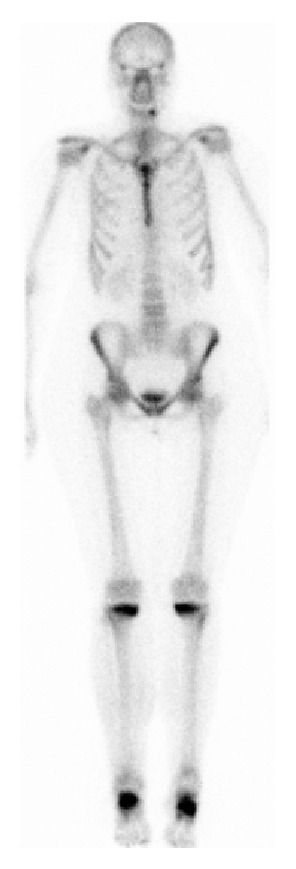


The preliminary diagnosis was a bilateral insufficiency fracture of the proximal tibia after a minor trauma and the use of methotrexate. The bone scintigraphy showed increased activity on the medial sides of the proximal tibias and in both hands and feet, matching the symptoms of her rheumatoid arthritis. The bone scintigraphy showed no evidence of malignancy (Figure 3). Furthermore, a bone densitometry was performed to determine and display possible osteoporosis. Normal bone density of the femur was seen (*T*-score: −0.8 SD, *Z*-score: −0.2 SD). In the lumbar spine, according to the WHO classification, osteopenia was found (*T*-score: −1.7 SD, *Z*-score: −0.8 SD). Laboratory results of the patient showed no abnormalities (e.g., serum calcium 2.52 mmol/L and alkaline phosphatase 130 U/L).

### 2.1. Therapeutic Considerations

The goal of treatment is to reduce pain and improve mobilization. Cast immobilization can provide an analgesic effect; however, our patient was not able to mobilize with crutches because of her rheumatic hands. She was therefore advised to continue mobilization, which would have a beneficial effect on the rheumatoid arthritis. Immobilization would reduce muscle mass and increase stiffness of the knee, which will increase pain and limitations as a result.

Intermittent parathyroid hormone (iPTH) is the only approved therapy that increases bone formation by osteoblasts and can be used in cases of bone loss due to conditions such as osteoporosis [3]. In this case it could be considered as a therapy to stimulate fracture healing.

In consultation with the rheumatologist, it was decided to stop MTX treatment. There are cases described where fracture healing and pain regression occurred after the MTX was stopped [4]. However, one must be careful to attribute this positive effect to the discontinuation of the drug, as fracture healing is dependent on multiple factors. The patient was asked to report when the symptoms of rheumatoid arthritis would increase after withdrawal of the MTX.

After two months, the pain regressed significantly and walking difficulties were reduced. On the X-rays callus formation was visible, which revealed that the fracture was healed (Figure 4).

## 3. Discussion

Fractures associated with MTX use were first reported in 1970 in children with acute leukemia treated with high doses [5]. Elevated calcium levels were found in urine and faeces, suggesting increased bone resorption. Symptoms included osteoporosis, bone pain, and insufficiency fractures, especially at the level of the distal tibia. High concentrations of MTX were found in the synovial membrane and in cortical and trabecular bone [6]. When the drug was stopped pain reduced and the fracture healed. In vitro studies showed an inhibitory effect of MTX on osteoblast proliferation [7]. No evident loss of bone density is seen in patients with rheumatoid arthritis [8]. However, if patients are treated concomitantly with prednisone, greater loss of bone density is seen in the lumbar spine than in those treated with the same dose of corticosteroids without MTX [9]. These differences can be explained by a dissimilarity in dose, treatment duration, follow-up, additional medication, and underlying disease. The role of MTX in the etiology of fractures remains unclear. There are often multiple risk factors for fractures, such as disease activity, immobilization, or corticosteroid use.

The presentation of knee injuries can be misleading, as this case described. It is unclear whether or not the problems are caused by the use of MTX. Multiple risk factors for insufficiency fractures are at play. The pain had an acute onset after a minimal trauma. The patient had been immobile for a week because of the pain. Reduced mobility increases the risk of insufficiency fractures. It is described that chronic use of low-dose corticosteroids can also negatively affect the bone metabolism and increase the risk of fractures [10]. Rheumatoid arthritis can cause local and systemic osteoporosis and abnormal bone turnover [11]. However, there was no evidence of osteoporosis in this case. This reduces the likelihood of an osteoporotic fracture and increases the presumption that MTX plays a role in these fractures. However, a sharp contrast remains between the small number of patients with insufficiency fractures and the large number of patients treated with MTX. This conflicting information creates a dilemma for the physician. Withdrawal of the MTX may cause a flare-up of rheumatoid arthritis with adverse consequences for the patients. When frequently controlled, withdrawal of the drug seems to be a legitimate choice because it has proven to have a beneficial effect on fracture healing.

## 4. Conclusion

In case of an insufficiency fracture conventional X-rays may show no abnormalities. MRI or bone scintigraphy can prove the diagnosis. Insufficiency fractures are more common in patients with joint diseases such as rheumatoid arthritis. MTX is a possible risk factor for insufficiency fractures in patients with rheumatoid arthritis, although a causal link between methotrexate and insufficiency fractures is debatable. Methotrexate osteopathy should be considered when people treated with MTX indicate pain in the tibia. Withdrawal of the drug may be the best option in case of a proven fracture.

## Lessons

- Methotrexate (MTX) is a drug of first choice in the treatment of inflammatory joint disorders such as rheumatoid arthritis and psoriatic arthritis.
- When people who use MTX localize pain in the tibia MTX osteopathy should be considered.
- The drug may be responsible for insufficiency fractures in rheumatoid arthritis because of low bone turnover due to osteoblast inhibition. However, its role remains debatable due to multiple risk factors for fractures, such as disease activity, immobilization, or corticosteroid use.
- Magnetic resonance imaging or bone scintigraphy may be conclusive if conventional X-rays show no abnormalities.
- It is advisable to stop MTX therapy to enhance fracture healing.

## Figures and Tables

**Figure 4:**